# Supplementary material for: Genetic and mechanistic basis for APOBEC3H alternative splicing, retrovirus restriction, and counteraction by HIV-1 protease
Source: Nat Commun. 2018 Oct 8;9:4137. doi: 10.1038/s41467-018-06594-3 (PMC6175962; doi:10.1038/s41467-018-06594-3)
Supplement: Supplementary file 1 — Supplementary Information [file 41467_2018_6594_MOESM1_ESM.pdf]

Supplementary Information for:

Genetic and mechanistic basis for APOBEC3H alternative splicing, retrovirus restriction, and counteraction by HIV-1 protease

Diako Ebrahimi[1,#], Christopher M. Richards[1,#], Michael A. Carpenter[1,2], Jiayi Wang[1], Terumasa Ikeda[1], Jordan T. Becker[1], Adam Z. Cheng[1], Jennifer L. McCann[1], Nadine M. Shaban[1], Daniel J. Salamango[1], Gabriel J. Starrett[1,4], Jairam R. Lingappa[3], Jeongsik Yong[1], William L. Brown[1], and Reuben S. Harris[1,2,*]

[1] Department of Biochemistry, Molecular Biology and Biophysics, Masonic Cancer Center, Institute for Molecular Virology, Center for Genome Engineering, University of Minnesota, Minneapolis, MN, USA, 55455

[2] Howard Hughes Medical Institute, University of Minnesota, Minneapolis, MN, USA, 55455

[3] Departments of Global Health, Medicine and Pediatrics, University of Washington, Seattle, WA, USA, 98104

[4] Present address: Lab of Cellular Oncology, Center for Cancer Research, National Cancer Institutes of Health, Bethesda, MD 20892, USA

[#] Equal contributors

[*] Correspondence: rsh@umn.edu

Author e-mail addresses: diako@umn.edu, rich1081@umn.edu, mcarpent@umn.edu, wang2284@umn.edu, tikeda@umn.edu, beck1169@umn.edu, cheng396@umn.edu, mccan121@umn.edu, nmshaban@umn.edu, dsalaman@umn.edu, gjstarrett@gmail.com, lingappa@uw.edu, jyong@umn.edu, brown344@umn.edu, rsh@umn.edu

Supplement information: 1 supplementary table (below), 6 supplementary figures (below), and one large data set (separately included as “Supplementary Data”).

Supplementary Table 1. DNA oligonucleotides used in this study.

Site-directed mutagenesis primers (5'-to-3')

G184A_for	CTGCGCGCGCACGGCCGGAATCTTTAT
G184A_rev	ATAAAGATTCCGGCCGTGCGCGCGCAG
V185A_for	CCTGCGCGCGCGCCCGGAATC
V185A_rev	GATTCCGGGCGCGCGCGCAGG
R186A_for	GGCCCTGCGCGGCCACGCCCGGAA
R186A_rev	TTCCGGGCGTGGCCGCGCAGGGCC
Q188A_for	CATATAGCGGCCCGCCGCGCGCACGCCC
Q188A_rev	GGGCGTGCGCGCGGCGGGCCGCTATATG
G189A_for	CCATATAGCGGGCCTGCGCGCGC
G189A_rev	GCGCGCGCAGGCCCGCTATATGG
R190A_for	CAGAATATCCATATAGGCGCCCTGCGCGCGCAC
R190A_rev	GTGCGCGCGCAGGGCGCCTATATGGATATTCTG
V185Q_for	GCCCTGCGCGCGCTGGCCCGGAATCTTT
V185Q_rev	AAAGATTCCGGGCCAGCGCGCGCAGGGC
V185W_for	GCCCTGCGCGCGCCAGCCCGGAATCTTT
V185W_rev	AAAGATTCCGGGCTGGCGCGCGCAGGGC
V185E_for	CCTGCGCGCGCTCGCCCGGAATC
V185E_rev	GATTCCGGGCGAGCGCGCGCAGG
R186W_for	CGGCCCTGCGCCCACACGCCCGGAA
R186W_rev	TTCCGGGCGTGTGGGCGCAGGGCCG
R186E_for	AGCGGCCCTGCGCCTCCACGCCCGGAATC
R186E_rev	GATTCCGGGCGTGGAGGCGCAGGGCCGCT
R186H_for	GCCCTGCGCGTGCACGCCCGG
R186H_rev	CCGGGCGTGCACGCGCAGGGC
A187Q_for	AGCGGCCCTGCTGGCGCACGCCCG
A187Q_rev	CGGGCGTGCGCCAGCAGGGCCGCT
A187W_for	AGCGGCCCTGCCAGCGCACGCCCG
A187W_rev	CGGGCGTGCGCTGGCAGGGCCGCT
A187E_for	GCGGCCCTGCTCGCGCACGCC
A187E_rev	GGCGTGCGCGAGCAGGGCCGC
A187H_for	CATATAGCGGCCCTGATGGCGCACGCCCGGAAT
A187H_rev	ATTCCGGGCGTGCGCCATCAGGGCCGCTATATG
A187R_for	AGCGGCCCTGCCTGCGCACGCCCG
A187R_rev	CGGGCGTGCGCAGGCAGGGCCGCT
VR185186AA_for	CGGCCCTGCGCGGCCGCGCCCGGAATCT
VR185186AA_rev	AGATTCCGGGCGCGGCCGCGCAGGGCCG
RA186187EE_for	CCATATAGCGGCCCTGCTCCTCCACGCCCGGAATCTTTA
RA186187EE_rev	TAAAGATTCCGGGCGTGGAGGAGCAGGGCCGCTATATGG

V185X_for	GCCCTGCGCGCGCTAGCCCGGAATCTTT
V185X_rev	AAAGATTCCGGGCTAGCGCGCGCAGGGC
R186X_for	ATAGCGGCCCTGCGCCTACACGCCCGGAATCTT
R186X_rev	AAGATTCCGGGCGTGTAGGCGCAGGGCCGCTAT
A187X_for	TAGCGGCCCTGCTAGCGCACGCCCGG
A187X_rev	CCGGGCGTGCGCTAGCAGGGCCGCTA
188X_for	ATAGCGGCCCTACGCGCGCACGC
188X_rev	GCGTGCGCGCGTAGGGCCGCTAT
pZ3618Msdm_for	TTGGCTTTCTTTGAAATATACATATGCTACTACACTAAACTA TTCCATGTTCTGATCC
pZ3618Msdm_rev	GGATCAGAACATGGAATAGTTTAGTGTAGTAGCATATGTAT ATTTCAAAGAAAGCCAA
p3618Fsdm_for	TGGCTTTCCTTGAAATATACATATGCTACTACACTAAACTAT TCCATGTTCTGATC
p3618Fsdm_rev	GATCAGAACATGGAATAGTTTAGTGTAGTAGCATATGTATAT TTCAAGGAAAGCCA

PCR primers (5’-to-3’)

pZ3618M_for	CCCTACAATCCCCAAAGTCA
pZ3618M_rev	GCCTATTCTGCTATGTTGACACCCAATTCTGAA
p3618F_for	CCAAAGTCAGGGAGTAGTAGAATCC
p3618F_rev	GCCTATTCTGCTATGTTGACACCCAATTCTGAA

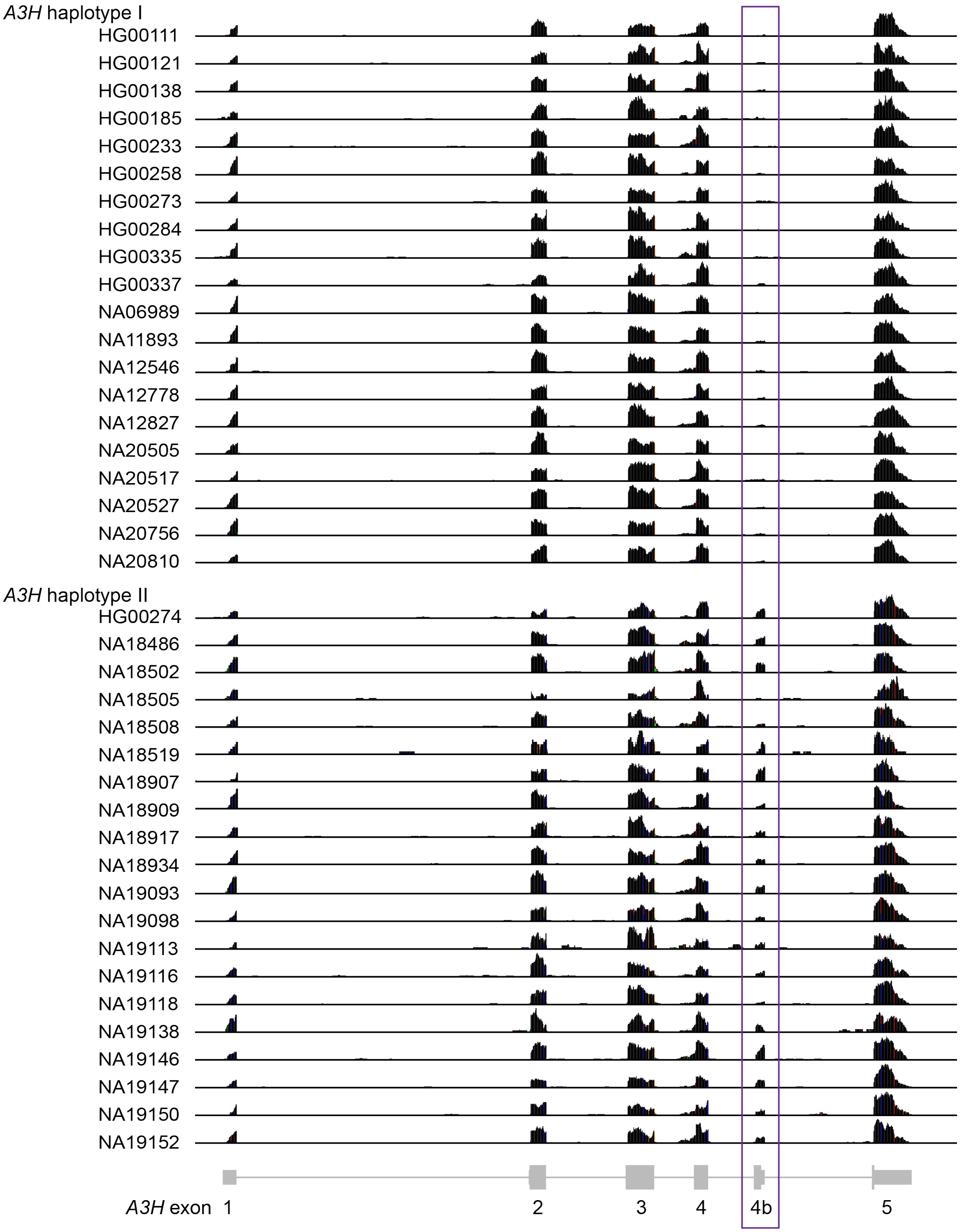

Supplementary Fig. 1. Differential splicing of *A3H* haplotypes I and II. Representative RNAseq profiles from LCLs derived from individuals homozygous for *A3H* haplotypes I or II with the exon 4b region boxed (n=20 per genotype). Codes correspond to 1000 Genomes Project donor identification numbers.

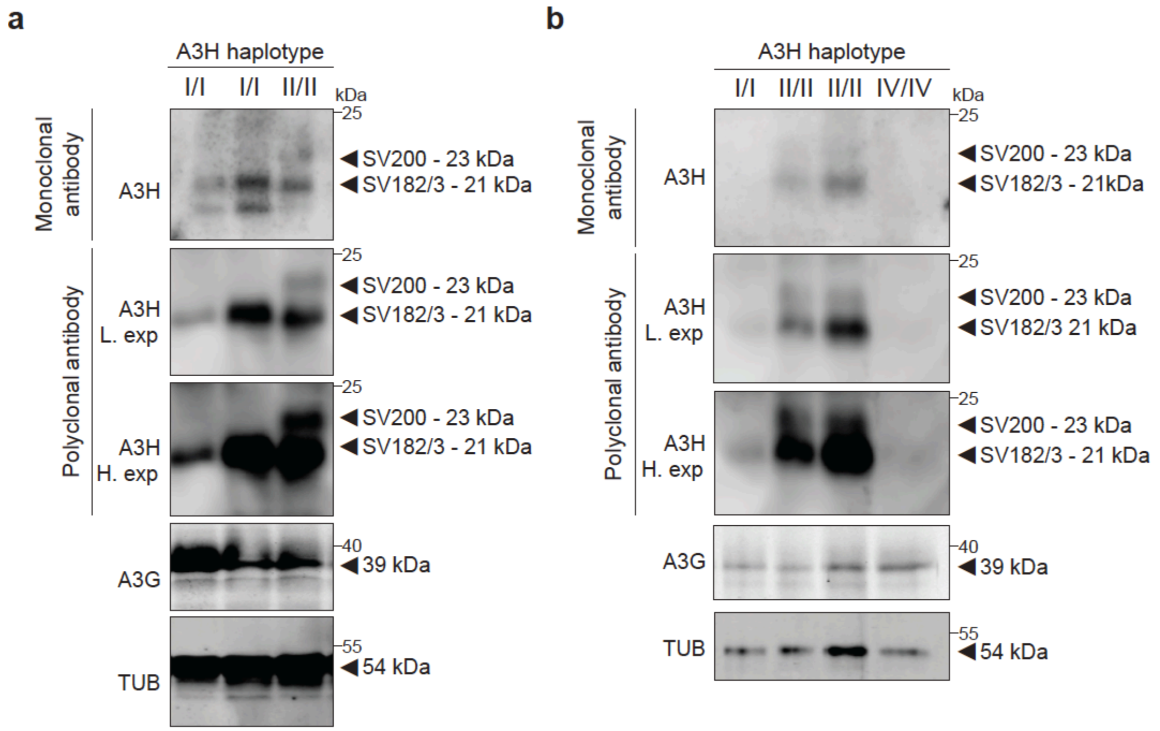

Supplementary Fig. 2. A3H SV200 is expressed specifically in cells with haplotype II genotypes.
a, **b**, Immunoblots of LCL (a) or PBMC (b) whole cell extracts derived from individuals with the indicated *A3H* haplotypes. Low and high exposures are provided for anti-A3H blots to show SV200 specificity for the haplotype II genotype. The upper blot was probed with a mouse anti-human A3H mAb P1D8-1 and the lower with a commercial rabbit anti-human A3H pAb. Anti-A3G and anti-TUBULIN (TUB) blots are shown as controls.

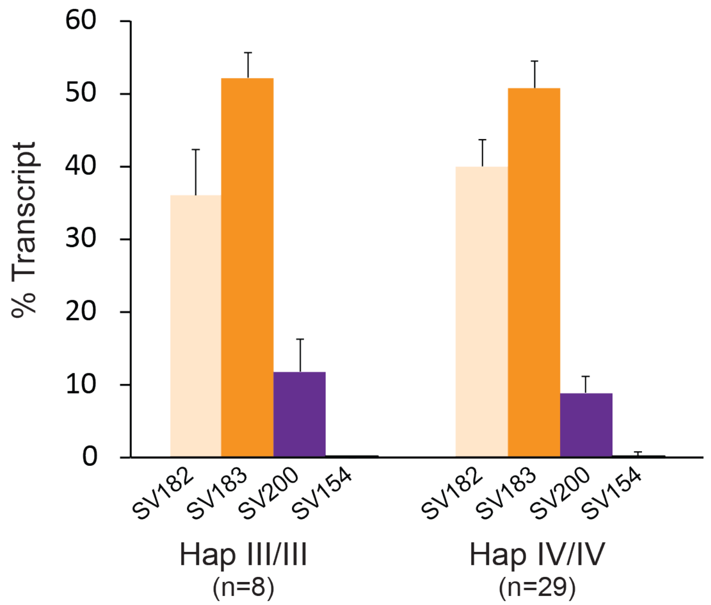

Supplementary Fig. 3. Differential *A3H* SV200 expression is unlikely due to coding variants R105, D121, or D178.
Bar charts reporting the average percentage of each splice variant observed in LCLs from donors with the indicated *A3H* haplotype (n values are shown and error bars represent 95% CI).

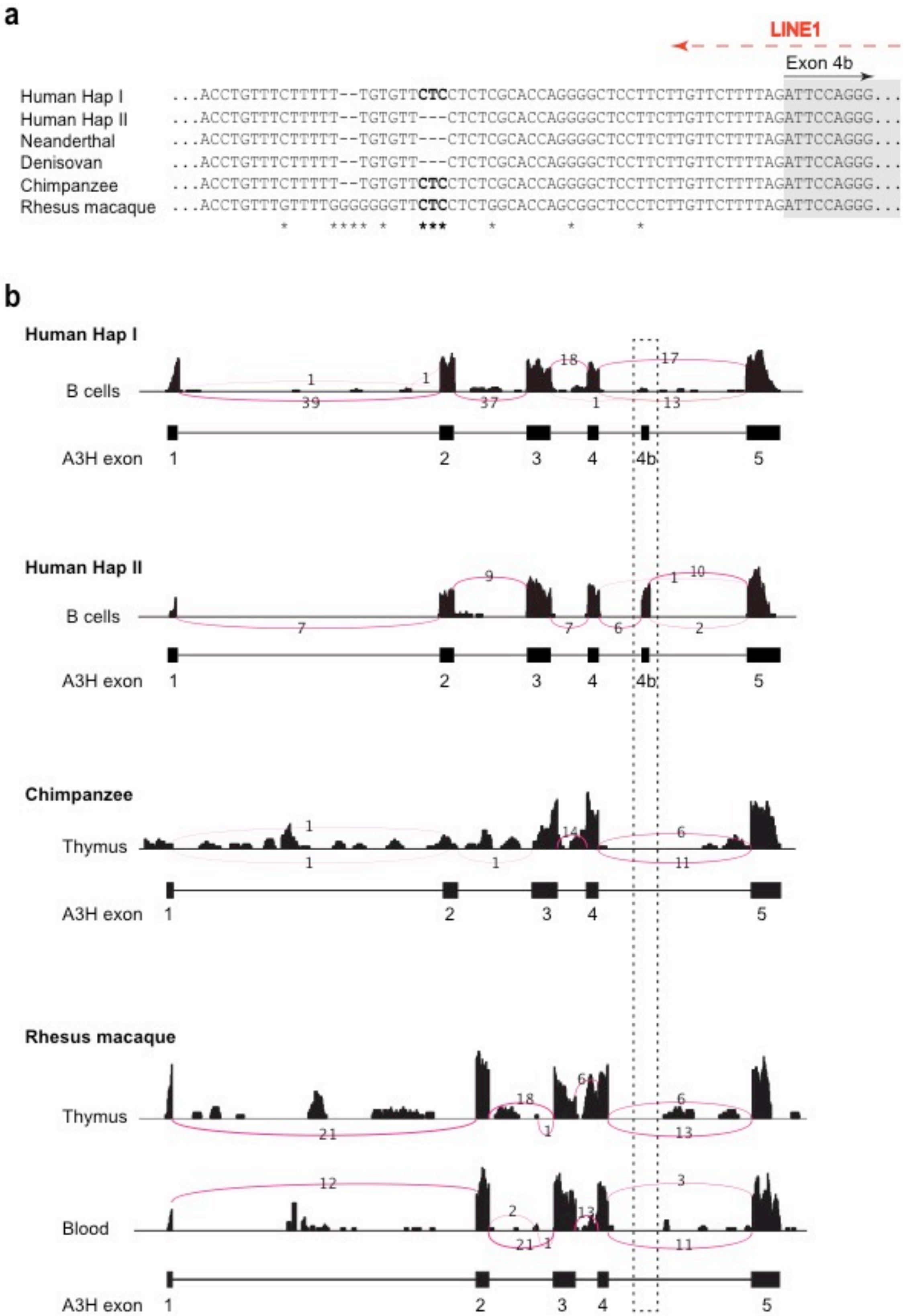
a
LINE1
Exon 4b
Human Hap I ...ACCTGTTTCTTTTT--TGTGTTCTCCTCTCGCACCAGGGGCTCCTTCTTGTTCTTTTAGATTCCAGGG...
Human Hap II ...ACCTGTTTCTTTTT--TGTGTT---CTCTCGCACCAGGGGCTCCTTCTTGTTCTTTTAGATTCCAGGG...
Neanderthal ...ACCTGTTTCTTTTT--TGTGTT---CTCTCGCACCAGGGGCTCCTTCTTGTTCTTTTAGATTCCAGGG...
Denisovan ...ACCTGTTTCTTTTT--TGTGTT---CTCTCGCACCAGGGGCTCCTTCTTGTTCTTTTAGATTCCAGGG...
Chimpanzee ...ACCTGTTTCTTTTT--TGTGTTCTCCTCTCGCACCAGGGGCTCCTTCTTGTTCTTTTAGATTCCAGGG...
Rhesus macaque ...ACCTGTTTGTTTTGGGGGGGTTCTCCTCTGGCACCAGCGGCTCCCTCTTGTTCTTTTAGATTCCAGGG...
b
Human Hap I
B cells
A3H exon
1 2 3 4 4b 5
Human Hap II
B cells
A3H exon
1 2 3 4 4b 5
Chimpanzee
Thymus
A3H exon
1 2 3 4 5
Rhesus macaque
Thymus
Blood
A3H exon
1 2 3 4 5

Supplementary Fig. 4. Genomic and transcriptomic data for humans, archaics, and representative non-human primates.

a, An alignment of the intron 4 genomic DNA region that harbors the ctc/Δctc variation. Only present day *A3H* haplotype II humans and archaic hominids, Neanderthals and Denisovans, share the Δctc variant suggesting a common molecular origin. Alternatively, the deletion allele may have occurred independently in archaic hominids and in ancestors to modern *A3H* haplotype II humans.

b, Representative *A3H* RNAseq profiles of the indicated cell or tissue type from humans with haplotype I or haplotype II genotypes, Neanderthals, Denisovans, chimpanzees, and rhesus macaques. The exon 4b region is boxed to highlight its expression in human cells with the hap II genotype but not in samples from the chimpanzee or rhesus macaque. Archaic hominid sequences were inferred from the Neanderthal and Denisovan tracks of the UCSC Genome Browser. The RNAseq of chimpanzee and rhesus macaque tissues were obtained from the Nonhuman Primate Reference Transcriptome Resource (NHPRTR, [www.nhprtr.org]). The raw fastq files were mapped to the chimpanzee reference genome (PanTro5) and rhesus macaque reference genome (rheMac8), respectively. The Sashimi plots were generated using the Integrative Genomics Viewer (IGV) program.

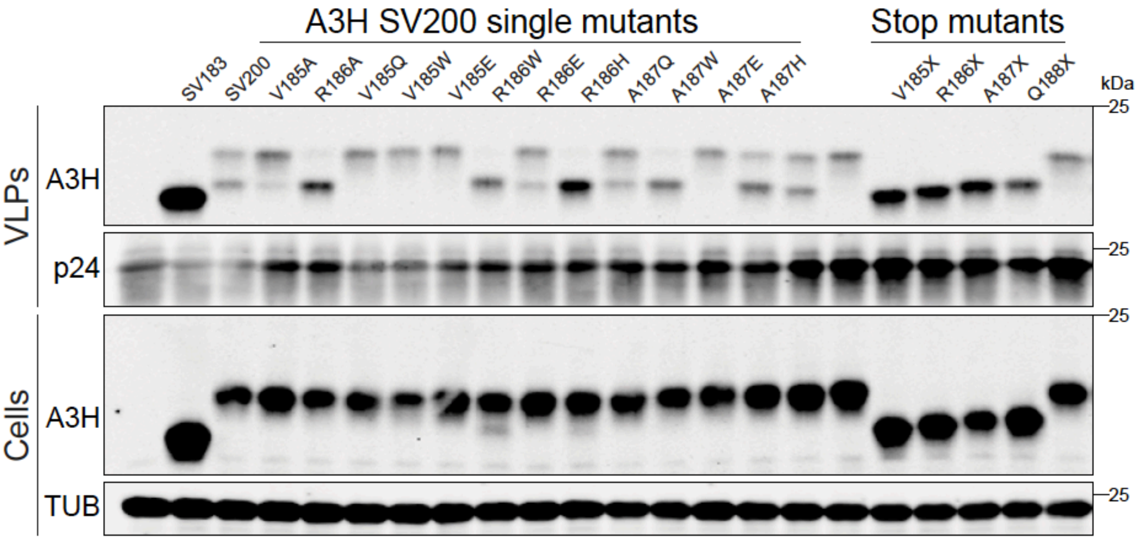

Supplementary Fig. 5. Mutation analysis of the HIV-1 protease cleavage site within the C-terminal end of A3H haplotype II SV200.
Immunoblots of A3H haplotype II SV200 and mutant derivatives in VLPs harvested from virus-producing 293T cells. The left/central portions of the blots show data for the indicated single amino acid substitution mutations, and the right region shows data for mutants with premature stop codons (X). Gag (p24) is shown as a VLP loading control. Corresponding 293T producer cell blots are shown below probed with antibodies against A3H and TUBULIN (TUB).

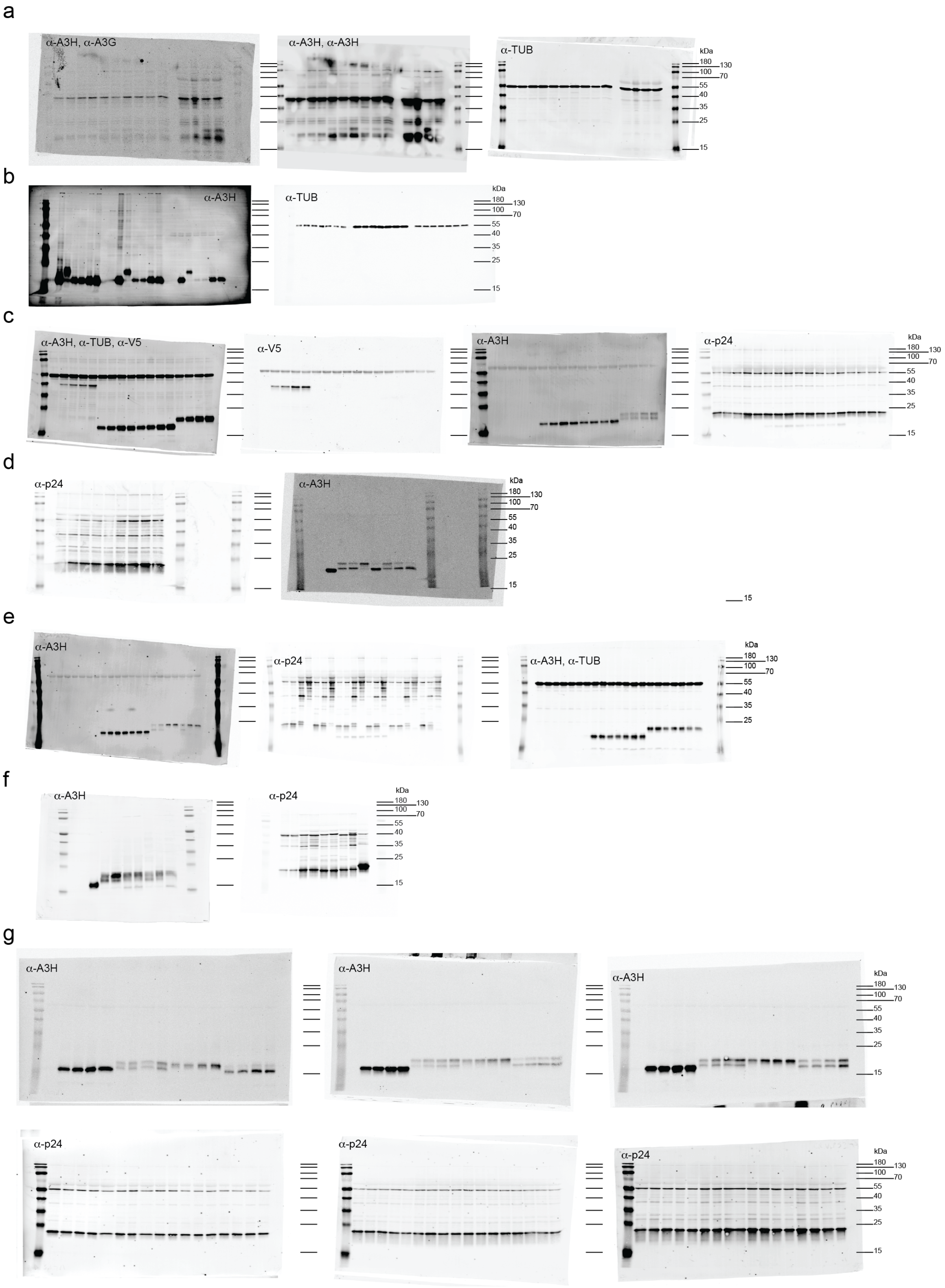
a
α-A3H, α-A3G
α-A3H, α-A3H
α-TUB
kDa
180
130
100
70
55
40
35
25
15
b
α-A3H
α-TUB
c
α-A3H, α-TUB, α-V5
α-V5
α-A3H
α-p24
d
α-p24
α-A3H
e
α-A3H
α-p24
α-A3H, α-TUB
f
α-A3H
α-p24
g
α-A3H
α-A3H
α-A3H
α-p24
α-p24
α-p24

Supplementary Fig. 6. Uncropped immunoblot images.
a, Immunoblots associated with **Fig. 2d-e**.
b, Immunoblots associated with **Fig. 4b**.
c, Immunoblots associated with **Fig. 5a**.
d, Immunoblots associated with **Fig. 5b**.
e, Immunoblots associated with **Fig. 5c**.
f, Immunoblots associated with **Fig. 5d**.
g, Immunoblots associated with **Fig. 6b**.
