# Supplementary material for: Genetic and mechanistic basis for APOBEC3H alternative splicing, retrovirus restriction, and counteraction by HIV-1 protease
Source: Nat Commun. 2018 Oct 8;9:4137. doi: 10.1038/s41467-018-06594-3 (PMC6175962; doi:10.1038/s41467-018-06594-3)
Supplement: Supplementary file 3 — Description of Additional Supplementary Files [file 41467_2018_6594_MOESM3_ESM.pdf]

Description of Additional Supplementary Files

File Name: Supplementary Data 1
Description: SNPs associated with A3H haplotype II SV200
